# Influence of Insect-Based Diets on Rural Leisure, Tourism, and Public Consumption—A Case Study from Yunnan, China

**DOI:** 10.3390/insects15110890

**Published:** 2024-11-14

**Authors:** Hsiao-Hsien Lin, Qi-Yuan Li, Ming-Hui Wang, Zhong-Xuan Jin, Chih-Chien Shen, Gan-Hong Chen

**Affiliations:** 1 School of Physical Education, Jiaying University, Meizhou 514015, China; chrishome12001@yahoo.com.tw (H.-H.L.); 18794721585@163.com (Q.-Y.L.); jinzhongxuan1209@163.com (Z.-X.J.); 2 Department of Healthcare Industry Technology Development and Management, National Chin-Yi University of Technology, Taichung 411030, Taiwan; 3 Department of Recreation and Sports Management, Tajen University, Pingtung 90741, Taiwan; 4 Department of Environmental Engineering, Daye University, Changhua 515006, Taiwan; ming703081@yahoo.com.tw; 5 National Shui-Li Vocational High School of Commerce and Industry, Nantou County 553308, Taiwan; 6 Institute of Physical Education and Health, Yulin Normal University, Yulin 537000, China; 7 School of Finance and Business, Guangdong Meizhou Vocational and Technical College, Meizhou 514011, China

**Keywords:** insect food, gourmet tourism, economic value, rural economy, sustainable development

## Abstract

This study analyzed the effects of insect-based diets on rural leisure activities, tourism, and consumption. A questionnaire survey was conducted, with 900 valid samples being obtained. The collected data were then analyzed through descriptive statistical analysis, a *t*-test, and a Pearson’s product-moment correlation coefficient test by using the SPSS 26.0 statistical software. Subsequently, semistructured interviews were conducted to collect expert and public opinions on the analysis results. Finally, literature findings and the collected questionnaire and interview data were reviewed through multivariate validation analysis. According to the results, the public agrees that entomophagy culture and insect cuisine can enhance leisure and tourism appeal. Entomophagy culture can also stimulate rural economic, social, and environmental development as well as rural consumption. However, modern public infrastructure and medical facilities are still required to increase tourism accessibility. Local governments must improve public sanitation, moral standards, and the overall quality of tourism and living environments. They must also attract corporate investment and cultivate entrepreneurship among residents to develop new insect-based tourism and leisure activities, food, and products. These measures can enhance the influence of insects on the sustainable development of leisure activities, tourism, and economies in rural regions.

## Simple Summary

This study examined the potential of insect-based diets or products for developing rural leisure tourism. The results indicate that insect-based diets or products can help revitalize the economy but are insufficient on their own to drive economic revitalization and stimulate consumption. Instead, improving public infrastructure and tourist facilities, expanding consumer options, and increasing the quality of tourism services and medical care are key for enhancing the use of insect-based diets or goods to stimulate leisure activities, tourism, and consumer engagement.

## 1. Introduction

Tourism has been a key industry for economic development in various countries since the late 19th century [1]. Key tourism features, including ethnic culture, historical monuments, and ecological resources, are promoted by governments worldwide as leisure attractions [2,3]. Ethnic and culinary cultural tourism refers to a category of tourism activities based on the inherent culture, customs, and culinary traditions of an ethnic group or region; such tourism is aimed at attracting visitors to explore dining experiences, travel, and leisure activities related to these distinct cultural features [4,5]. The province of Yunnan, located in southwestern China, is mainly characterized by its highland terrain [6]. Chinese historical records indicate that ethnic minorities in Yunnan are prone to conflicts because of livelihood, economic, and political factors [7,8,9,10]. Furthermore, the mostly plateau terrain in Yunnan imposes constraints that limit external transportation [11]. These factors collectively reduce the time and space available for agriculture, limit foreign trade opportunities [12], and contribute to food shortages or food security problems [13]. However, because of the abundance of forests and freshwater resources in Yunnan, the ecology of the region is diverse, allowing for the production of diverse edible resources, including various animal and plant species [14,15]. In Yunnan, local communities utilize local ecological resources to collect food materials to address their food shortages [12,16,17]. Although insects are not fully edible and the hygiene of their natural environment is uncertain, they generally contain high levels of protein, fats, lipids, vitamins, and minerals. Moreover, insects are adaptable, reproduce quickly, and can be easily collected in large quantities [18,19,20,21]. When handled and cooked appropriately, insects can serve as a source of basic nutrition and a temporary food substitute, reducing food waste and contributing to food security [22,23]. Consequently, insects have become a main food source for meeting nutrition needs among various ethnic groups in Yunnan [6,12,24,25,26,27,28,29,30,31,32].

Documented records indicate that the local culture of consuming insects in Yunnan spans more than 3000 years. To date, approximately 707,700 people from 22 ethnic minorities may still uphold an insect-based diet culture [25]. Since 2015, the local government has been promoting Yunnan’s entomophagy culture as an aspect of the region’s cultural tourism. Currently, at least 24 themed restaurants in Yunnan offer insect-based specialty dishes prepared using straightforward cooking techniques [26,27]. Insect availability peaks in summer; however, with advancements in food processing technology, insect consumers and the general public can use refrigeration or freezing techniques to preserve the integrity and freshness of insects. These methods enable the consumption of insects year-round, regardless of the season. During the peak tourist season each year, entomophagy culture can attract up to 1,399,300 tourists in Yunnan, creating business opportunities valued at approximately USD 253.6 million [28]. Furthermore, insects have long contributed to advancements in agriculture, horticulture, biology, chemistry, and the medical and beauty industries [6,16,30,31,32,33], helping governments to obtain sufficient funding for improving public infrastructure, such as local transportation, leisure, and entertainment, while increasing personal incomes and supporting quality-of-life improvements [34,35,36,37,38]. This trend reflects the growing human reliance on insects, highlighting the potential for developing insect-related leisure and food tourism activities that foster economic development.

However, some scholars have argued that policy objectives, expectations, established theories, and experiences are often influenced by factors such as time, space, and target demographics [2,3,26], leading to positive and negative outcomes that may deviate from initial expectations [36,37]. These outcomes require time to manifest fully [3,26,39], and they must be corroborated by the public or further evidence before clear and accurate conclusions can be obtained [39,40]. Tseng et al. suggested that collecting insights from residents and tourists can enable a more in-depth assessment of the effectiveness of local policies, the identification of problems [38,39,40], the acquisition of objective findings, and the development of appropriate recommendations [3,26,39]. Some of the latest studies on insect-related topics focused on the exploration of agricultural promotion [31,41], insect feed production and waste decomposition [16,42], insect breeding techniques and nutritional value [21,30,43], insect food safety and marketing [30,44,45,46], the contribution of insect-based foods to local economic development [41,42,45,47,48,49,50], ethnic and traditional food culture [5,16,46,47,51], and folklore and medicine [16,52]. However, not many studies have examined (or expand) the effects of insect-based food culture or products on rural leisure activities, tourism economic development, and consumption intention from the perspective of various stakeholders (e.g., ethnic groups that consume insects as food) or in the context of specific regions (e.g., Yunnan). Therefore, this study aims to address research gaps while validating the impact of insect-based diets on rural recreation, tourism, and public consumption.

## 2. Literature Review

### 2.1. Effects of Insects, Human Food and Leisure, and Tourism on Economic Development

Insects can serve as a key source of nutrition for humans. Insects cooked using various techniques have become staple foods across numerous countries, ethnic groups, or resource-constrained communities [18,19,20,21,22,23,24,25,26,27,28,29,30,31,32]. Despite the traditional appeal of insect-based foods, their simple cooking methods and special appearance have caused a gradual decrease in their popularity in some countries with an entomophagy culture. Nevertheless, insects are a viable source of food because of their ease of capture and high abundance [18,19,20,21]. When leisure patterns change and tourism grows, tourists’ desire to explore novel experiences gradually increases [38,39,40]. Remote areas tend to be economically underdeveloped, and local governments and communities can address this challenge by establishing tourist attractions to revitalize the local economy. In this context, distinct culinary features, such as insect dishes, can be a draw for tourists [26,27,38,39,40]. Furthermore, the existing reliance on insects in various areas [16,25,26,27,30,31,32,33], including agriculture, horticulture, biology, chemistry, medicine, beauty, leisure, entertainment, and tourism, reflects the usefulness of insects. Therefore, insects can potentially be used to promote leisure activities, economic growth, and consumer interest in rural areas.

### 2.2. Willingness to Travel and Spend

Willingness is a behavioral quality or state that reflects people’s interest in an activity, such as visiting an attraction, acquiring an experience, or purchasing a product or service; this behavioral quality influences their subsequent actions [53,54]. Willingness to travel [55] includes revisiting intention [56]. In the present study, willingness to travel refers to an individual’s intention to travel again to a location to consume insect-based foods or engage in entomophagy culture.

Some scholars have argued that the likelihood of people engaging in tourism consumption can be an indicator of future tourism decisions [37,57]. In particular, the willingness to revisit can be used to evaluate people’s attitudes toward the consumption of tourism activities or goods [56], confirm the effectiveness of local development, and assess the feasibility of sustainable tourism development in coastal villages [3]. When tourist destinations and villages become more developed, people’s willingness to travel to these areas increases, which in turn promotes tourism development, particularly in river-adjacent villages [57]. Therefore, in the present study, the willingness to travel was used as a variable to understand the current effectiveness of rural tourism development in river-adjacent villages, thereby enabling an exploration of the effects of rural food culture on the leisure and economic development of river-adjacent rural areas.

### 2.3. Effects of Economic, Social, Environmental, and Ecological Development on River-Adjacent Rural Areas

Tourism development is mainly based on the distinct characteristics of a tourism destination’s local and surrounding tourism resources, which attract tourists to visit and spend money. Although tourism development can affect the development of the ecological environment along rivers [58], it can provide economic benefits to local communities [3], enhance the visibility and cultural characteristics of villages [59], and improve the local living environment.

Scholars have highlighted that tourism development influences 12 factors related to local economic development, such as industry and facilities [59]. Tourism development can also affect the quality of social services in 14 aspects of development, such as the commercialization of community buildings [3], and help address 10 challenges, such as improving village transportation facilities and living spaces [57]; however, it has adverse effects on the natural environment along river areas, including their ecology, water quality, vegetation, and woodlands [59]. These findings indicate that promoting tourism development has positive and negative effects on the local and surrounding ecology [37,60,61]. The effects of tourism can be assessed by considering various indicators and obtaining public perceptions, which help clarify how rural food culture influences the leisure and economic development of river-adjacent villages. Table 1 provides an overview of the effects of economic, social, environmental, and ecological development on villages.

## 3. Methods and Instruments

### 3.1. Research Framework

This study investigated the local entomophagy culture in Lijiang City, Yunnan, China, as a case study, focusing on the local community. The effects of rural food culture tourism on the development of the rural economy, river ecology, and human leisure activities in a high-risk environment were analyzed on the basis of theories of consumption intention and cognitive effects of rural economic, social, environmental, and ecological development.

**Figure 1:**
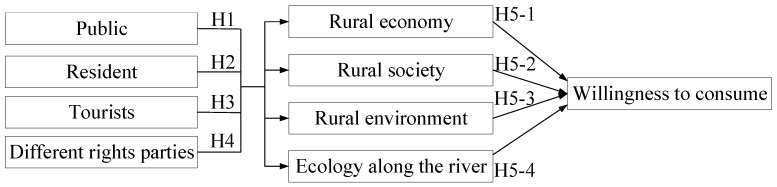
Research framework. H: Hypothesis.

A mixed-methods approach was adopted in the present study. First, literature on climate change, food crises, insect-based diets, rivers, rural development, and consumption intentions [7,8,9,10,11,12,13,14,15,16,17,18,19,20,21,22,23,24,25,26,27,28,29,30,31,32,33,34,35,36,37,38] was reviewed to identify relevant themes and directions. Subsequently, a questionnaire was developed and edited on the basis of literature on tourism development [36,37,38,39,40,54,55,56,57] and consumer preferences [3,58,59,60,61]. Three scholars were then invited to check the validity of the questionnaire content. The statistical software SPSS 26.0 was used to conduct exploratory factor analysis (EFA) to validate the reliability and validity of the questionnaire. Descriptive statistics were used to analyze the general perceptions of 900 respondents, with the perceptions of residents and tourists analyzed separately. A *t*-test was then conducted to analyze the perceptions of various stakeholders. Next, Pearson correlation analysis was performed to examine awareness of economic, social, environmental, and ecological development among the surveyed individuals. Thereafter, the opinions of eight individuals, comprising scholars, residents, and tourists, on the data analysis results were collected. Finally, multivariate analysis was conducted to provide a basis for discussion. The research framework is depicted in Figure 1.

### 3.2. Research Hypotheses

Tourism development is mainly based on the distinct characteristics of local and sur-rounding tourism resources, which attract tourists to visit and spend money [2,3]. When natural resources, local culture, art, monuments, and commodities have strong appeal, the potential for leisure engagement, tourism experiences, and consumer spending is enhanced [53,54,56]. Although most insects have sharp-looking and jointed limbs that are inedible, their bodies are nutritious. Furthermore, diverse insect species exist, and insects are abundant, capable of reproducing quickly, easy to collect, and distinct in appearance and taste when cooked [18,19,20,21,22,23,24,25,26,27,28,29,30,31,32]. These advantages make insects a viable source of food for humans and a product that can be developed and promoted through food and cultural tourism activities [38,39,40]. These advantageous attributes can encourage tourists and consumers to travel, consume, and engage in leisure activities, thereby increasing tourists’ consumption willingness [62,63] and revitalizing economic development in rural areas [26,27,38,39,40]. However, these outcomes require time to manifest [3,26,39] fully, and they must be corroborated by the public or further evidence before clear and accurate conclusions can be obtained [39,40]. Therefore, whether insect consumption can stimulate rural economic, social, environmental, and ecological development is not fully clear. Furthermore, the potential for insect consumption to increase consumption willingness remains uncertain. Accordingly, five hypotheses were formulated on the basis of the aforementioned literature review, and the validity of these hypotheses was tested.

The five formulated hypotheses are as follows:

**Hypothesis** **1 (H1):** *The public believes that insect consumption significantly influences rural economic, social, environmental, and ecological development.*

**Hypothesis** **2 (H2):** *Residents believe that insect consumption significantly influences rural economic, social, environmental, and ecological development*.

**Hypothesis** **3 (H3):** *Tourists believe that insect consumption significantly influences rural economic, social, environmental, and ecological development*.

**Hypothesis** **4 (H4):** *No significant difference exists among various stakeholders in terms of their perception of the effects of insect consumption on rural economic, social, environmental, and ecological development*.

**Hypothesis** **5 (H5):** *The current state of rural economic, social, environmental, and ecological development has no significant effect on consumer willingness*.

### 3.3. Research Process, Methodology, and Analysis

Scholars have highlighted that qualitative research is effective for capturing rich details and exploring personal experiences in depth [2,64], whereas quantitative research is suitable for processing large quantities of data and conducting statistical analysis [3,65]. Quantitative research can be employed to test hypotheses and draw conclusions; however, it cannot provide an in-depth analysis of underlying issues [66]. By contrast, performing qualitative research alone is time-consuming and the collected data cannot be quantified [67]. Through a mixed-methods research design, the advantages of both quantitative and qualitative research can be leveraged, addressing their respective shortcomings [2,3,68,69,70]. Therefore, literature on rural food and cultural tourism was first collected to understand the research topic. Subsequently, a questionnaire was designed on the basis of theoretical knowledge related to rural tourism, river tourism, and consumption intention. A total of 1000 questionnaires were distributed, with 900 valid samples being obtained (90% response rate). The collected data were analyzed using SPSS 26.0, with descriptive statistical analysis (average score and frequency distribution tests), a *t*-test, a Pearson product-moment correlation coefficient (PPMCC) test, and regression analysis being conducted. Frequency distribution was used to analyze the distribution of background variables in the sample, an average score test was conducted to analyze respondents’ perceptions, a *t*-test was performed to verify the differences in perception between residents and tourists, and a PPMCC test was employed to verify the mutual influence between independent and dependent variables. Moreover, open-ended interviews were conducted to collect respondents’ opinions on the data analysis results. Finally, multivariate analysis was performed to categorize, summarize, compare, and analyze all collected information [71,72].

### 3.4. Methods, Scope, Objectives, and Limitations

In the present study, various limitations, such as constraints related to labor, budget, time, transportation, interviewees’ personal understanding, and external environmental factors, affected the analysis of the questionnaire data and interview results. This research was limited to the Chinese province of Yunnan, focusing on the influence of insects on promoting leisure activities, economic development, and consumption willingness. Given the cultural and regional specificity of consumption behaviors, the results of this study might not be fully applicable to other countries or regions with different entomophagy cultures, food tourism cultures, and leisure economies. Future research can address this limitation to enhance the applicability of the results of the present study.

### 3.5. Ethical Considerations

Prior to conducting the formal survey, the research process was thoroughly explained to all participants. All interviews and data collection processes were performed only after signed consent forms were obtained from the participants. The study procedure adhered to the guidelines for public non-anonymous and interactive research, and all interventional research and participant data were deidentified. Legal notices outlined the study’s purpose, experimental procedures, and individual motor learning behavior assessments, emphasizing that these procedures would be conducted in a safe and legal teaching environment. All procedures posed minimal risk, with the probability or intensity of harm or physical discomfort during the experiment being lower than that experienced in daily life. The study protocol complied with the guidelines of the Chinese Department of Health (Executive Yuan Gazette No. 1010265075) [73] and adhered to the requirements of Articles 1004 and 1009 of the Civil Code of the People’s Republic of China [74]. The survey was conducted in accordance with legal, transparent, and fair procedures and in compliance with the Declaration of Helsinki.

### 3.6. Design of Research Tools

The questionnaire was divided into two main sections. The first section was used to collect the following background information: identity (resident or tourist), gender (male or female), and age (≤20, 21–30, 31–40, 41–50, 51–60, or ≥61 years). The second section focused on the following variables: consumer attractiveness, satisfaction, and consumption willingness. The questionnaire comprised 34 items, each of which was rated on a 5-point Likert scale ranging from 1 (*least agreeable*) to 5 (*most agreeable*) (Table 2). After the initial questionnaire was edited, three experts specializing in food, tourism, and marketing were invited to check the validity of the questionnaire content and confirm the relevance of the initial questionnaire themes. Moreover, five respondents representing industry professionals, residents, and tourists were invited to provide feedback on the questionnaire analysis results, which were then used as the basis for subsequent research and exploration (Table 2).

The three recruited experts confirmed that the topics of the preliminary questionnaire were suitable for analysis. The present study implemented a rigorous research process, obtained precise judgments to support inferences, and finally reached the most appropriate conclusions and recommendations [75]. The questionnaire was finalized in May 2021, and conceptual sampling was conducted to obtain 100 responses from residents and individuals with relevant travel experience. SPSS 26.0 was then used to conduct calculations and data testing. In data testing, a Kaiser–Meyer–Olkin (KMO) value of >0.06 and a *p* value of <0.01 in Bartlett’s test indicated that the scale was suitable for factor analysis [76,77]. Moreover, an *α* value of >0.60 was regarded as indicating high reliability, allowing for further analysis [70,75,76,77].

First, the cognitive dimension of rural economic development was analyzed. The results revealed a KMO value of >0.467, Bartlett’s approximate *χ*^2^ value of 120.109, a degree of freedom (df) value of 55, and a significance level of <0.001; thus, the scale was suitable for factor analysis. EFA was then conducted, revealing an interpretable variance of 73.25% and a total *α* value of 0.800, with the *α* value of each issue being >0.6 [70,75,76,77]. These results indicated that the developed scale was reliable, allowing for subsequent analysis. After the expert validity check and field survey, the 11 items for assessing rural economic development were retained.

Second, the cognitive dimension of rural social development was examined. The results revealed a KMO value of >0.521, Bartlett’s approximate *χ*^2^ value of 356.172, a df value of 78, and a significance level of <0.001. Thus, the scale was reliable, allowing for subsequent factor analysis. EFA was then conducted, revealing an interpretable variance of 74.48% and a total *α* value of 0.788, with the *α* value of each issue being >0.6 [70,75,76,77]. After the expert validity check and field survey, the 13 items for assessing rural social development were retained.

Third, the cognitive dimension of rural environmental development was investigated. The results revealed a KMO value of >0.509, Bartlett’s approximate *χ*^2^ value of 65.879, a df value of 21, and a significance level of <0.001. Thus, the scale was reliable, allowing for subsequent factor analysis. EFA was then conducted, revealing an interpretable variance of 71.66% and total *α* value of 0.780, with the *α* value of each issue being >0.6 [70,75,76,77]. After the expert validity check and field survey, the seven items for assessing rural environmental development were retained.

Fourth, the cognitive dimension of ecological development along the river was explored. The results revealed a KMO value of >0.544, Bartlett’s approximate *χ*^2^ value of 58.872, a df value of 15, and a significance level of <0.001. Thus, the scale was reliable, allowing for subsequent factor analysis. EFA was then conducted, revealing an interpretable variance of 66.628% and total α value of 0.788, with the *α* value of each issue being >0.6 [70,75,76,77]. After the expert validity check and field survey, the six items for assessing ecological development along the river were retained.

Finally, the dimension of consumption intention was analyzed. The results revealed a KMO value of >0.463, Bartlett’s approximate *χ*^2^ value of 1067.425, a df value of 3, and a significance level of <0.001. Thus, the scale was reliable, allowing for subsequent factor analysis. EFA was then conducted, revealing an interpretable variance of 82.37% and total *α* value of 0.886, with the α value of each issue being >0.6 [70,75,76,77]. After the expert validity check and field survey, the three items for assessing health consumption attractiveness were retained. Overall, the findings confirmed the reliability of the adopted scales for subsequent analyses (Table 3).

### 3.7. Analysis

#### 3.7.1. Background Analysis

The present study employed statistical methods to analyze the background information of 900 respondents. Among the respondents, 65.5% and 34.5% were residents and tourists, respectively; 61.1% were women; and those aged ≥20 years constituted the smallest age group (10.5%). In the sample, most respondents were residents with insect consumption experience, female, and aged between 41 and 50 years (Table 4).

#### 3.7.2. Public Awareness of the Current State of Economic, Social, Environmental, and Ecological Development

The perceptions of all 900 respondents regarding the current economic, social, environmental, and ecological development of rural areas were analyzed. As presented in Table 5, the highest-rated perceptions were reported for the following issues: improvement of medical standards (2.46), sufficient parking space or leisure facilities (2.46), increase in waste on water surfaces (2.46), and maintenance of historical scenery and sites (2.35). By contrast, the lowest-rated perceptions were reported for the following issues: waste pollution affecting the local environment (2.27), increase in oil pollution on water resources (2.20), loss of local architectural features (2.10), and increase in salary levels (2.09).

#### 3.7.3. Residents’ Awareness of Current Economic, Social, Environmental, and Ecological Development

Residents’ perceptions of the current economic, social, environmental, and ecological development of rural areas were analyzed. As presented in Table 5, the highest-rated perceptions were reported for the following issues: increased parking space or forest waste (2.50), a sufficient number of public toilets (2.48), sufficient leisure facilities (2.47), and improved medical standards (2.45). By contrast, the lowest-rated perceptions were reported for the following issues: increase in oil pollution on water resources (2.29), well-maintained historical scenery and sites (2.22), increased tourism-related construction and integration of local characteristics into industries (2.14), and loss of local architectural features (2.01).

#### 3.7.4. Tourists’ Awareness of Economic, Social, Environmental, and Ecological Development

Tourists’ perceptions of the current economic, social, environmental, and ecological development of rural areas were analyzed. As presented in Table 5, the highest-rated perceptions were reported for the following issues: increase in pollution of water by waste (2.53), improving medical standards (2.47), increasing willingness to participate in the community (2.45), and improved maintenance of historical scenery and sites (2.41). By contrast, the lowest-rated perceptions were reported for the following issues: a sufficient number of public toilets (2.23), increased oil pollution on water resources (2.17), friendly public interaction (2.05), and increased salary (2.04).

#### 3.7.5. Analysis of Differences Among Various Stakeholders in Terms of Their Awareness of Rural Economic, Social, Environmental, and Riverine Ecological Development

The differences in the awareness of various stakeholders regarding the current economic, social, environmental, and ecological development of rural areas were analyzed. As presented in Table 5, significant differences (*p* < 0.01) were identified among the stakeholders in terms of their awareness regarding the following issues: increased industrial development, integration of local characteristics into industries, improving medical standards, increasing people’s willingness to participate in the community, promoting the development of private tourism organizations, increasing the willingness of youths to return to their hometowns, a sufficient number of public toilets, and overdevelopment of land. Among these issues, residents reported the highest perception ratings for the following issues: promoting the development of private tourism organizations, a sufficient number of public toilets, and overdevelopment of land. By contrast, tourists reported the highest perception ratings for the following issues: increased industrial development, integration of local characteristics into industries, improving medical standards, promoting the development of private tourism organizations, and increasing the willingness of youths to return to their hometowns.

#### 3.7.6. Analysis of the Effects of Rural Economic, Social, Environmental, and Ecological Development on Consumption Intention

The effects of rural economic, social, environmental, and ecological development on consumption intention were analyzed. Social development had positive and significant effects (*p* > 0.01) on consumption intention, recommendations to family and friends, and consumer awareness. Environmental development had negative and significant effects on consumption intention, recommendations to family and friends, and reconsumption awareness (*p* > 0.01). Moreover, ecological development had positive and significant effects on consumption intention, reconsumption intention, recommendations to family and friends, and the sharing of consumption experiences (*p* > 0.01). By contrast, economic development did not have a significant effect on consumption intention (*p* > 0.01).

**Figure 2:**
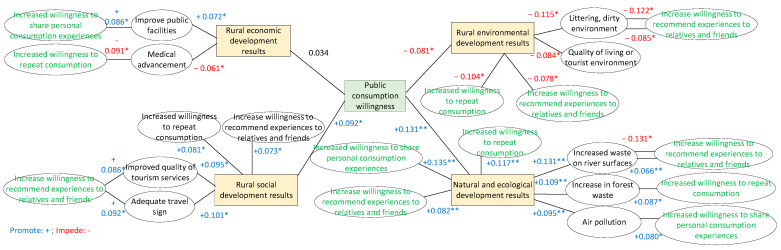
Correlations of economic, social, environmental, and ecological development of rural areas with consumption intention. * *p* < 0.05; ** *p* < 0.01.

Notably, public facilities had significant effects on economic development and the sharing of consumption experiences (*p* > 0.01), and improved medical care had significant effects on economic development and awareness of reconsumption (*p* > 0.01). Service quality had significant effects on social development and recommendations to family and friends (*p* > 0.01), and adequate travel labels had significant effects on social development and recommendations to family and friends (*p* > 0.01). Moreover, tourism waste had a significant effect on environmental development and recommendations to family and friends (*p* > 0.01), and damage to the village environment had significant effects on environmental development and recommendations to family and friends (*p* > 0.01). As displayed in Figure 2, water surface waste had significant effects on ecological development and recommendations to family and friends (*p* > 0.01); forest waste had significant effects on ecological development and recommendations to family and friends (*p* > 0.01); and air pollution had significant effects on ecological effects, willingness to reconsume, and sharing of consumption experiences (*p* > 0.01).

## 4. Discussion

### 4.1. Public Awareness of the Current State of Rural Economic, Social, Environmental, and Ecological Development

Although not all parts of insects are edible, they still have considerable culinary, nutritional, and medicinal value [5,16,21,30,31,41,43,46,47,51]. In addition to providing consumer value, entomophagy culture has leisure and tourism appeal [5,16,25,26,27,28,46,47,51], and it can contribute to the development of technologies, medicine, and economies. Insects can serve as a stable food source for people lacking access to nutritious food. They can also provide tourists with a unique food culture experience as well as solutions for addressing medical problems or developing new products. These characteristics can help people to design new leisure and tourism activities that take advantage of the benefits of insects to stimulate consumption and boost the local economy. Therefore, the public perceives that insects can be used in rural areas to promote the leisure and tourism industries; improve medical care, leisure, parking, and recreational facilities; and raise awareness of the conservation of historical sites and cultural relics. However, because of the lack of public infrastructure, tourism services, and management personnel, environmental awareness among tourists is low, and the cost of environmental maintenance has increased, thereby limiting the appeal of consumption activities. Modern tourist facilities are limited, and water pollution and waste management remain challenging. The aforementioned problems contribute to the public perception that although insect-related leisure and tourism hold potential for economic development, improvements in public infrastructure and leisure and tourism facilities must be achieved to fully realize the benefits of insect-related activities. These results are inconsistent with findings in the literature [3,11,22,27,28,29,30] and do not support H1.

Addressing outdated public infrastructure, expanding leisure and tourism facilities, and improving the quality of tourist services and environmental sanitation can strengthen the effects of insect-related activities on economic development and enhance public perceptions of the benefits of these activities.

### 4.2. Residents’ Awareness of the Current State of Rural Economic, Social, Environmental, and Ecological Development

Limitations related to ethnic culture, conflicts, topography, and transportation lead to food scarcity, lifestyle changes, and food safety crises, which can pose survival challenges [7,8,9,10,11,12,13]. Insects are easy to collect, abundant, and nutrient-dense [5,16,21,30,31,41,43,46,47,51], making them a promising option for communities affected by food insecurity. Insects can also contribute to the development of agriculture and medicine as well as leisure, tourism, and recreational activities tailored to regional cultural characteristics. These advantages are likely the main reasons why residents perceive insects as being useful for helping rural areas to create tourism business opportunities; improve government finances; support parking, public toilet, leisure, and medical facilities; and improve the conservation of historical buildings. However, rural areas face various challenges, including a shortage of young labor, limited entrepreneurial skills and capital among residents, and differences in public moral standards and awareness of environmental hygiene. These challenges have limited the quality of rural living standards and rural tourism, thereby reducing tourism appeal, tourists’ willingness to spend, and tourism-related economic benefits and industrial development. The aforementioned results are inconsistent with the literature [3,11,22,27,28,29,30,43] and do not support H2.

Local governments should encourage the retention of young labor, improve residents’ awareness of environmental hygiene, and enhance residents’ entrepreneurial skills and capital. A necessary step is to create new tourist attractions, maintain the quality of living and tourist environments, and improve the willingness of the public to spend money. These strategies can enhance the benefits of using insects for rural leisure, tourism, and economic development while increasing residents’ recognition of and confidence in the value of insects.

### 4.3. Tourists’ Awareness of the Current State of Economic, Social, Environmental, and Ecological Development

Insect-based foods exhibit a distinctive taste and appearance as well as high nutritional content [5,16,21,30,31,41,43,46,47,51], and governments have leveraged these attributes to create leisure and tourism attractions [5,16,25,26,27,28,46,47,51]. This strategy has successfully attracted people to travel and spend, driving local leisure and tourism industries and revitalizing local economies [25,26,27,28]. It also enables governments and entrepreneurs to increase their revenue and raise sufficient funds to improve the quality of local public infrastructure, medical care, and sanitation standards. However, despite the potential of insect-related tourism, problems such as a lack of young labor, insufficient public restroom facilities, low-quality tourism services, and poor environmental cleanliness may deter tourists from visiting relevant areas. The aforementioned findings are inconsistent with the literature [3,11,22,27,28,29,30,44] and do not support H3.

Local governments should increase wages for tourism-related jobs, actively attract young workers, expand public restroom facilities, and improve the quality of living and tourism environments. These measures can enhance the benefits of using insects for rural leisure, tourism, and economic development while increasing tourists’ recognition of the value of insects and their willingness to spend.

### 4.4. Differences in the Awareness of Various Stakeholders Regarding the Current State of Rural Economic, Social, Environmental and Ecological Development

Insects can help meet the nutritional needs of humans; preserve food culture; promote recreational activities, tourism, and consumer experiences; and stimulate economic development [5,16,25,26,27,28,46,47,51]. They can also provide financial benefits for governments, helping them to raise funds to improve public infrastructure and medical care. Tourists expect that leisure- and tourism-related activities, commodities, and industries incorporating insects as tourism elements should be diversified; they also expect that stable and high-quality tourism services should be provided to meet leisure and travel demands. By contrast, residents value the development of local public facilities, high community involvement, and attracting youths to return to their hometowns to create business opportunities. These differences between tourists and residents have led to divergent views among stakeholders on aspects such as the development of creative industries, public infrastructure, medical facilities, land development, and new tourism organizations.

Challenges remain in making insect-related tourism more appealing, including the low attractiveness of such tourism, the lack of leisure and tourism-related options, the lack of tourism facilities and organizations, and the poor quality of tourism services and environmental sanitation. These challenges have impeded public support and reduced the desire of tourists to consume. The aforementioned problems have prevented tourists from recognizing the potential of insects for rural leisure and economic development while eroding their confidence in local tourism public facilities, service quality, and policy planning.

Local residents’ practice of consuming insects has deep cultural roots, and their entrepreneurial skills and knowledge are limited. Thus, a high level of similarity and lack of variety are observed in local foods and local dishes. Moreover, public tourism facilities are outdated, transportation is inconvenient, entomophagy culture is not widespread, and the market and consumer base for insect-based products are small. These drawbacks affect foreign investment in tourism related to entomophagy culture and the willingness of youths to return to their hometowns for employment and development. Consequently, residents are less likely to recognize insects as viable contributors to rural leisure and economic growth. They are also disappointed in insufficient government efforts to recruit youths to return to their hometowns to increase labor availability, open up markets, stimulate business and employment opportunities.

In summary, although the public acknowledges the nutritional value of insects and agrees that they can benefit economic development, key challenges limit the attractiveness of insect consumption, including the low quality of public construction, leisure facilities, and tourism services; the lack of options for insect-related industries and commodities; and unsanitary living and tourism environments. Residents expect insects to be used to promote leisure and tourism industries and revitalize the local economy and infrastructure. By contrast, tourists expect rural villages to use insects to create leisure and tourism attractions to meet their needs with respect to local tourism consumption. Residents and tourists have divergent views on the quality of local tourism infrastructure, restrooms, medical facilities, tourism enterprise performance, entrepreneurship and employment opportunities, and the likelihood of youths returning to their hometowns. Therefore, different stakeholders hold different opinions on the effects of using insects to promote rural leisure, tourism, economy, and consumption. These findings differ from the literature [2,3,13,14,15,16,17,18,19,20,43] and do not support H4.

### 4.5. Correlations of Rural Economic, Social, Environmental, and Ecological Development with Consumer Willingness

Insects have nutritional value and can support advancements in agriculture and health care; they can also serve as an attraction in the context of leisure and tourism activities [5,16,25,26,27,28,46,47,51]. These attributes not only provide solutions to short-term food shortages and health-care challenges but also enhance public awareness of insect consumption. The practice of consuming insects and the distinct characteristics of insect foods, such as their appearance and taste, can also attract tourists, stimulate public interest in travel and consumption, and generate consumer flow and business opportunities. This practice encourages the public to explore insect consumption, thereby enhancing their positive experiences and willingness to consume. Local governments and businesses can benefit economically by raising funds to improve urban development or business operations. Improvements in rural infrastructure, corporate image, and service quality contribute to a positive tourism experience and reinforce the willingness to consume. Therefore, when rural communities become more developed, consumers become more likely to consume, make recommendations to family and friends, and reconsume. Furthermore, when ecological protection and development are enhanced, the willingness to consume increases.

Entomophagy culture exhibits distinct regional and national elements [25,26,27,28]. However, tourism development can alter natural landscapes, damage historical buildings, and generate waste and air and water pollution. These problems can result in negative public perceptions of insect consumption, reduce positive perceptions of leisure and tourism activities based on entomophagy culture, and reduce the willingness to consume. Thus, enhancing rural economic development and reducing environmental harm increase the desire to consume and the likelihood of consumers making recommendations to their family and friends.

Insects can be used to generate tourism revenue and promote rural economic development. To enhance these benefits, efforts must be made to improve public infrastructure, tourism facilities, and service quality; harness advanced medical technology; and create safe, comfortable, and sanitary living and tourism environments. These elements collectively enhance the public’s positive travel experiences and increase their willingness to consume. Moreover, their desire to reconsume increases. The aforementioned measures constitute the optimal model for governments, enterprises, and individuals to emphasize the benefits of entomophagy culture, raise investment capital, improve infrastructure, and develop insect-based leisure activities, tourism, and commodities, thereby achieving the goal of sustainable development. Therefore, improving economic growth increases the willingness of the public to share their consumption experiences, improving the standard of health care enhances economic development and the desire to consume, improving the quality of services facilitates social development and increases the willingness to make recommendations to family and friends, and improving the planning of tourism facilities enhances people’s ability to socialize and their desire to recommend their family and friends to spend.

Finally, entomophagy culture can attract tourists and generate substantial economic benefits, thereby revitalizing rural leisure, tourism, and economic development [3,58,59,60,61]. However, leisure activities, tourism, and consumption produce tourism waste, and the overexploitation of natural resources (e.g., land, forests, and vegetation) to meet tourism demand has emerged as a problem. Empty houses, water pollution, and floating waste, all of which damage the ecology and living environment, are also caused by tourism-related transportation. These problems have resulted in negative public perceptions of tourism experiences, particularly in the context of entomophagy culture within rural areas. Therefore, problems related to excessive tourism waste, environmental damage to villages, water pollution, and excessive forest waste reduce tourists’ willingness to make recommendations to family and friends. Finally, air pollution decreases tourists’ willingness to reconsume and share experiences.

## 5. Conclusions and Recommendations

Insects, with their nutritional benefits and distinct appeal, can help supplement human nutritional needs while enhancing tourism attractions. They can promote rural leisure activities, tourism, and economic development and stimulate consumer interest. Both residents and tourists widely recognize the value of insects. However, local communities must overcome several social challenges, including population aging, a lack of young workers, and limited employment opportunities, to reap the benefits of insect-based tourism activities. Few young people are willing to return to their hometowns for career or entrepreneurial development. Moreover, the lack of modern public facilities, poor health-care conditions, limited entrepreneurial skills among business owners, and the limited variety of tourism products and consumer options must be addressed. This situation is exacerbated by insufficient labor, poor tourism and residential environment management, and low service quality. These problems reduce the public’s positive perceptions of the value of insects and affect their willingness to consume, hindering the use of insects to promote leisure tourism and economic development. Consequently, stimulating consumer interest in insect-based tourism activities or products becomes difficult.

Specifically, the present study used the Yunnan province of China as a case study to ex-amine the effects of insect consumption by individuals on rural leisure activities, tourism, economic development, and consumption willingness. The results of the present study reveal the disruptive effect of such consumption on the aforementioned factors. They can also increase government awareness of ethnic minority culture (e.g., entomophagy culture) and improve public perceptions of entomophagy. Ultimately, the results can help governments and people to understand the potential of unique local entomophagy cultures and insect resources to contribute to leisure activities, tourism, and economic development.

Given the aforementioned findings, recommendations are formulated for governments and industries. These recommendations are detailed in the following text.

### 5.1. Recommendations for Governments

Local governments should actively improve public infrastructure, develop tourism and medical facilities, enhance public awareness of environmental literacy, increase the number of employed service personnel, and maintain a clean environment. Establishing a youth development fund to attract a young workforce is also crucial. These measures can help rejuvenate the workforce for industries and services, increase tourism appeal, and enhance consumer confidence.

### 5.2. Recommendations for Industries

Local entomophagy entrepreneurs should utilize local resources to develop new products; employ older artisans with artistic, cultural, and culinary skills; and invite foreign talent to engage in skill exchanges. Another essential measure is to expand the recruitment of young talents to increase the pool of young service workers and industry vitality. These actions can strengthen tourism appeal and service quality while increasing the public’s positive tourism experiences with entomophagy culture.

### 5.3. Future Research Directions

The results of the present study are limited by cost, labor, material resources, topics, target population, scope, and various logistical challenges. Future researchers can increase their sample size, adopt diverse research methods, explore new topics, and expand the scope of the survey by including additional countries, regions, or demographic segments.

## Figures and Tables

**Table 1:** Overview of the effects of economic, social, environmental, and ecological development on villages.

Aspects	Topics
Village economy	Industries, facilities, employment, wages, consumption, construction, prices, health, leisure, culture and creativity, incentives, and policy coordination.
Village society	Service quality, commercialization of community buildings, community development participation, tourism indicators, recreational facilities, community environment, Indigenous cultures, vocational training opportunities, living environment, youth development, citizen interaction, preservation of traditional cultures, community security and safety, and cultural industry investment.
Village environment	Transportation facilities, living space, distribution of tourist waste, tourist building areas, parking, and rest facilities.
Riverine ecology	Ecological environment, water quality, vegetation, and woodlands.

**Table 2:** Analysis of interviewees’ backgrounds and questionnaire results.

**Interviewees’ Backgrounds and Introduction to Interview Topics**						
**Identity**		**Gender**	**Duration of Residence (Years)/Number of Years of Work Experience**	**Identity**	**Gender**	**Duration of Residence (years)/Number of Years of Work Experience**
Professor		Male	18	Resident	Male	60
Professor		Female	15	Resident	Female	55
Professor		Male	9	Tourist	Male	32
Food entrepreneur		Female	22	Tourist	Female	47
Construct	Issues					
Effect on tourism development	Which aspect of insect-based foods or food culture do you think has the greatest effect on rural economic, social, environmental, and ecological development? Regarding the data analysis results, what do you think explains the effect of insect-based foods or food culture on rural economic, social, environmental, and ecological development? Please express your opinion and suggest possible contributing factors. What do you think residents or tourists will benefit the most from when insect-based foods or food culture is used to drive the economy and improve the current social, environmental, and ecological development? What reasons do residents or tourists have for thinking that economic, social, environmental, and ecological development might be disrupted? Please express your opinion and suggest possible contributing factors. Do you think that promoting insect-based foods or food culture in rural areas to drive the economy and improve current social, environmental, and ecological development will affect consumption willingness? Regarding the data analysis results, what are the reasons affecting consumption willingness? Please express your opinion and suggest possible contributing factors.					

**Table 3:** Analysis of the questionnaire for assessing travel attractiveness, satisfaction, and willingness to travel.

Construct (*α*)	Issues (*α*)
Effect of economic development (0.800)	Increase in tourism-related construction (0.781), increase in industrial development (0.783), integration of local characteristics into industries (0.786), improvement in public facility maintenance (0.782), increase in entrepreneurship and employment opportunities (0.754), increase in salary levels, increase in housing prices (8.00), improvement in public transportation (0.782), increase in medical standards (0.785), improvement in development protection policies (0.784), and development of creative goods (0.788).
Effect of social development (0.788)	Improved service quality (0.726), improved activity quality (0.740), increased leisure opportunities (0.770), increased willingness of people to participate in the community (0.720), adequate tourism signs (0.768), promotion of the development of private tourism organizations (0.788), increased willingness of youths to return to their hometowns (0.782), preservation of local culture or historical scenery (0.782), loss of local architectural features (0.780), friendly public interaction (0.782), adequate security personnel (0.780), and adequate parking space or rest facilities (0.784).
Effect of environmental development (0.780)	Quality of the village environment is affected (0.724), improved transportation accessibility (0.760), local environment is polluted by waste (0.780), overflowing tourism waste (0.780), sufficient availability of public waste bins (0.750), well-maintained historical scenery and monuments (0.760), and sufficient availability of public restrooms (0.768).
Effect of ecological development (0.788)	Increase in pollution of water by waste (0.780), increase in forest waste (0.788), increase in oil pollution of water resources (0.788), overdevelopment of land (0.780), habitat destruction (0.784), and severe air pollution (0.786).
Consumer willingness (0.886)	Willingness to consume again (0.800), sharing of consumption experiences (0.880), and recommending friends and relatives to consume (0.886).

**Table 4:** Background information on the questionnaire respondents.

Issue		%	Issue		%
Identity	resident	65.5%	Gender	male	38.9%
	Tourist	34.5%		female	61.1%
Age	Under 20	12.5%	Age	41–50	30.6%
	21–30	29.5%		Over 51	14.0%
	31–40	13.4%			

**Table 5:** Analysis of respondents’ perceptions regarding rural economic, social, environmental, and ecological development.

Construct	Issues	Public		Residents		Tourists		Different Rights Holders		
		Mean	Rank	Mean	Rank	Mean	Rank	Residents (SD)	Tourists (SD)	*p*-Value
Effect of economic development	Increase in tourism-related construction	2.21	6	2.14	10	2.24	6	1.110	1.108	0.626
	Increase in industrial development	2.26	5	2.17	9	2.30	5	0.957	1.151	0.000 **
	Integratio of local characteristics into industries	2.15	7	2.14	10	2.15	7	1.054	1.139	0.008 **
	Public facilities are well maintained	2.13	8	2.33	5	2.05	9	0.943	0.995	0.402
	Increase in entrepreneurship and employment opportunities	2.15	7	2.30	6	2.10	8	1.115	1.112	0.774
	Increase in salary and income	2.09	9	2.21	8	2.04	10	1.080	1.054	0.051
	Increase in housing prices	2.29	4	2.24	7	2.31	4	1.084	1.076	0.359
	Improved public transportation	2.40	2	2.38	4	2.40	2	1.079	1.017	0.313
	Improved medical standards	2.46	1	2.45	1	2.47	1	1.218	1.060	0.002 **
	Improved development and protection policies	2.40	2	2.39	3	2.40	2	1.076	0.991	0.052
	Development of creative products	2.36	3	2.42	2	2.34	4	1.094	1.141	0.483
Effect of social development	Improved service quality	2.25	7	2.29	9	2.23	8	1.050	1.108	0.195
	Improved activity quality	2.31	5	2.33	8	2.30	6	0.990	1.014	0.600
	Increased leisure opportunities	2.35	4	2.34	7	2.36	3	0.992	1.091	0.046
	Increased willingness to participate in the community	2.43	2	2.36	5	2.45	1	1.023	1.175	0.002 **
	Adequate tourism signage	2.43	2	2.44	3	2.43	2	0.962	1.034	0.425
	Promotion of the development of private tourism organizations	2.24	8	2.29	9	2.22	9	1.156	0.954	0.000 **
	Increased willingness of youths to return to their hometowns	2.26	6	2.07	10	2.33	5	1.041	1.178	0.001 **
	Preservation of local cultural or historical scenery	2.31	5	2.41	4	2.27	7	1.293	1.217	0.064
	Loss of local architectural features	2.10	10	2.01	11	2.13	10	0.975	1.000	0.202
	Friendly interaction with the public	2.13	9	2.35	6	2.05	11	1.067	1.083	0.426
	Adequate security personnel	2.42	3	2.45	2	2.41	3	0.987	1.040	0.311
	Sufficient parking space or resting facilities	2.46	1	2.47	1	2.45	1	0.992	1.033	0.426
Effect of environmental development	Quality of the village environment is affected	2.29	5	2.39	3	2.25	4	0.999	1.040	0.715
	Transportation accessibility is improved	2.34	2	2.36	4	2.33	2	1.142	1.181	0.360
	Local environment is polluted by waste	2.27	6	2.31	5	2.25	4	1.088	1.087	0.809
	Overflowing tourism waste	2.30	4	2.39	3	2.27	3	1.087	1.050	0.324
	Sufficient availability of public waste bins	2.32	3	2.46	2	2.27	3	0.983	1.099	0.026
	Well-maintained historical scenery and monuments	2.35	1	2.22	6	2.41	1	1.022	1.092	0.064
	Sufficient availability of public restrooms	2.30	4	2.48	1	2.23	5	0.968	1.127	0.000 **
Effect of ecological development	Increase in pollution of water by waste	2.46	1	2.30	5	2.53		1.059	1.096	0.994
	Increase in forest waste	2.29	5	2.50	1	2.21	5	0.972	1.069	0.324
	Increase in oil pollution on water resources	2.20	6	2.29	6	2.17	6	1.007	0.992	0.713
	Overexploitation of land	2.39	3	2.41	3	2.37	3	0.972	1.134	0.007 **
	Habitat destruction	2.43	2	2.32	4	2.47	2	1.059	1.129	0.024
	Severe air pollution	2.38	4	2.48	2	2.34	4	1.004	1.060	0.204

*p* < 0.01 **.

## Data Availability

The raw data supporting the conclusions of this article will be made available by the authors on request.
